# Quantum-Resistant Security Technologies for Resource-Constrained Edge Devices: Challenges and Lightweight Countermeasures

**DOI:** 10.3390/s26185859

**Published:** 2026-09-16

**Authors:** Fengsheng Zeng, Bahari Idrus, Mohammad Faidzul Nasrudin, Eddie Shahril Ismail

**Affiliations:** 1 School of Artificial Intelligence and Data Science, Yang-En University, Quanzhou 362000, China; 2 Center for Artificial Intelligence Technology, Faculty of Information Science and Technology, Universiti Kebangsaan Malaysia, Bangi 43600, Selangor, Malaysia; mfn@ukm.edu.my; 3 Department of Mathematical Sciences, Faculty of Science and Technology, Universiti Kebangsaan Malaysia, Bangi 43600, Selangor, Malaysia; esbi@ukm.edu.my

**Keywords:** Internet of Things (IoT) edge computing, quantum-resistant cryptography, quantum key distribution, resource constraints, lightweight encryption

## Abstract

With the rapid advancement of quantum computing technology, IoT edge devices widely deployed in industrial settings are facing severe threats to their quantum-resistant security. This work focuses on the challenges of adapting Quantum Key Distribution (QKD) and Post-Quantum Cryptography (PQC) for resource-constrained environments. This work systematically identifies four critical obstacles: difficulties in physical integration due to device heterogeneity; constraints imposed by the limited computing power and storage resources of edge nodes on PQC algorithm execution; compatibility gaps between QKD/PQC and existing industrial protocols (such as Modbus and Profinet); and the engineering cost pressures associated with large-scale deployment. To address these issues, the paper proposes three synergistic lightweight countermeasures: (1) a tiered deployment architecture for quantum-safe security that accommodates device heterogeneity, enabling dynamic trade-offs between security strength and resource overhead; (2) middleware for quantum-classical hybrid network protocol coordination, ensuring seamless integration between QKD/PQC and industrial control protocols; and (3) a three-tier distributed key management mechanism (edge node, network proxy, and central system) that offloads computation- and storage-intensive tasks to high-resource nodes, thereby alleviating bottlenecks at the edge. Experimental validation within a smart manufacturing factory’s sensor network demonstrates that the proposed scheme maintains post-quantum security capabilities (with QKD integration validated at the proof-of-concept level) while reducing per-node deployment costs by approximately 92.4% compared to pure QKD solutions and by about 32% compared to pure PQC solutions, all while facilitating seamless, non-disruptive upgrades. This study indicates that lightweight algorithm design, hardware-software co-optimization, and tiered architectural deployment constitute a viable pathway for advancing IoT edge security into the quantum-safe era.

## 1. Introduction

### 1.1. Research Background

Continuous breakthroughs in quantum computing technology are fundamentally undermining the security foundations of modern cryptographic systems. Shor’s algorithm is capable of breaking classical public-key cryptographic mechanisms based on large integer factorization and discrete logarithm problems (such as RSA and ECC) in polynomial time, which serve as the cornerstone of secure internet communication [1,2]. Even more critical is the “Harvest Now, Decrypt Later” attack model, which implies that attackers can collect encrypted data today and decrypt it in the future once large-scale, fault-tolerant quantum computers become available [1]. Should this threat materialize, critical sectors underpinned by traditional public-key infrastructure—such as the Industrial Internet of Things (IIoT), smart grids, and autonomous driving—face systemic security risks.

Meanwhile, the deep integration of the Internet of Things (IoT) and edge computing is reshaping the architectural landscape of industrial systems. It is projected that the global number of IoT devices will rise to 41.6 billion by 2025 [3]. A vast number of these are resource-constrained edge nodes, such as sensors, actuators, and smart meters, which are characterized by limited RAM (tens of kilobytes) and low clock speeds (tens of megahertz). Relying on battery power, these devices are incapable of supporting traditional, computationally intensive security protocols and struggle to meet the higher resource demands of post-quantum cryptographic schemes. Striking a balance between resource constraints and quantum-resistant security has thus become an urgent challenge shared by both academia and industry.

### 1.2. Current State of Research

Post-Quantum Cryptography (PQC) is widely recognized as the primary technological approach for addressing the threats posed by quantum computing. On 13 August 2024, the U.S. National Institute of Standards and Technology (NIST) officially released the first three post-quantum cryptography standards: FIPS 203 (ML-KEM, a lattice-based key-encapsulation mechanism), FIPS 204 (ML-DSA, a lattice-based digital signature algorithm), and FIPS 205 (SLH-DSA, a hash-based signature scheme) [4]. NIST mandated that U.S. federal systems complete the migration to these standards by 2035. Subsequently, in 2025, NIST released the Initial Public Draft of FIPS 206 (FN-DSA, a FALCON-based lattice signature scheme), with the final standard expected in late 2026 or early 2027 [1,5,6]. The release of these standards marks the transition of PQC from the stage of academic research to that of engineering deployment.

Researchers have conducted extensive work on PQC implementations for embedded platforms. Botros et al. proposed a memory-efficient, high-speed implementation of Kyber on the ARM Cortex-M4; by optimizing Number Theoretic Transform (NTT) computations, they more than doubled the NTT processing speed and achieved an overall performance that was 18% faster than the implementation by the early submitter [7]. Abdulrahman et al. subsequently achieved even faster implementations of Kyber and Dilithium on the Cortex-M4 [8]. In 2026, Chhetri et al. published the first systematic benchmark of the NIST-standardized ML-KEM and ML-DSA algorithms on the ARM Cortex-M0+; they found that ML-KEM-512 required only 35.7 ms to complete a full key exchange on an RP2040 running at 133 MHz, with an energy consumption of approximately 2.83 mJ, making it 17 times faster than ECDH-P256 key agreement on the same platform [1]. Regarding hardware acceleration, FPGA-based implementations of ML-KEM have demonstrated strong performance, with complete SoC-level implementations achieving performance gains of 1.6 to 2.1 times [9].

Regarding the integration of QKD and PQC, researchers have proposed a layered architecture that deploys QKD on high-value backbone links while implementing PQC within field and edge networks. Hybrid cryptographic systems are considered an effective approach to addressing quantum threats [10]. However, existing research has paid limited systematic attention to the unique challenges faced by resource-constrained edge devices when deploying quantum-resistant schemes, or to the corresponding lightweight countermeasures.

### 1.3. Research Contributions and Paper Structure

The main contributions of this work are as follows: (1) It systematically identifies and analyzes four core obstacles to deploying quantum-resistant technologies on edge devices; (2) it proposes three synergistic lightweight countermeasures covering the dimensions of architectural design, protocol compatibility, and key management; and (3) it validates the effectiveness of the proposed scheme through experiments in real-world industrial scenarios.

The structure of this work is as follows: Section 2 introduces the technical background of QKD and PQC and analyzes their applicability to edge scenarios; Section 3 systematically details the four core challenges; Section 4 proposes three lightweight countermeasures; Section 5 validates the scheme’s effectiveness through experimental case studies; and Section 6 summarizes the paper and outlines future research directions.

## 2. Fundamentals of Quantum-Safe Technology and Analysis of Edge Applicability

### 2.1. Principles of Quantum Key Distribution (QKD)

Quantum Key Distribution (QKD) leverages fundamental principles of quantum mechanics, specifically the Heisenberg uncertainty principle and the no-cloning theorem, to achieve information-theoretically secure key distribution [11]. Taking the classic BB84 protocol as an example, the sender encodes random bits using four polarization states, while the receiver randomly selects measurement bases to perform measurements; the two parties then compare their basis choices over a public channel, discard mismatched results, and ultimately share a secure key.

The core advantage of QKD lies in the fact that its security is grounded in the laws of physics rather than relying on any computational assumptions. Any eavesdropping on the quantum channel inevitably introduces detectable disturbances, alerting the communicating parties. However, this advantage comes with stringent implementation requirements: it necessitates specialized hardware such as single-photon sources, superconducting detectors, and low-loss optical fibers, while transmission distance is constrained by fiber loss (typically limited to under 100 km) [12]. These hardware requirements pose significant physical integration challenges for deploying QKD on edge devices.

### 2.2. Post-Quantum Cryptography (PQC) Algorithm System

**Figure 1:**
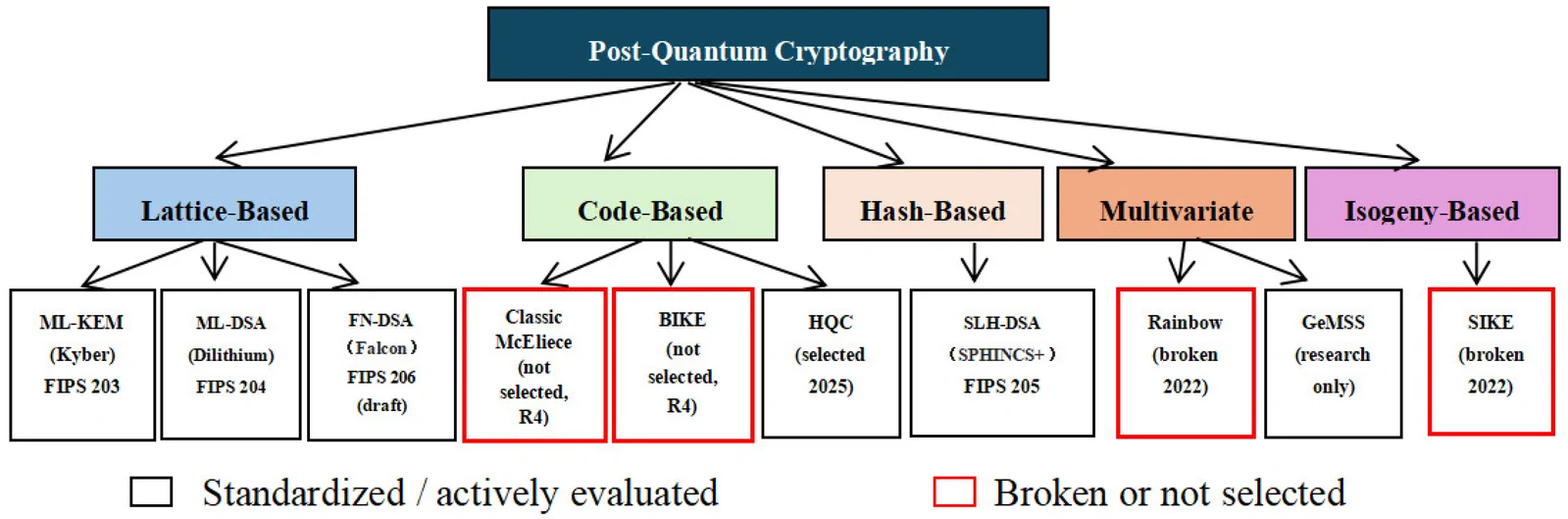
Taxonomy of post-quantum cryptography (Source: Adapted from NIST, 2024).

Unlike QKD, which relies on dedicated physical devices, PQC refers to mathematical cryptographic algorithms that can run on classical computers and resist quantum computer attacks. The mathematical foundations of PQC are diverse, mainly including families such as lattice cryptography, code cryptography, hash cryptography, multivariate cryptography, and homologous cryptography [13]. Among various PQC schemes, lattice cryptography has received the most attention due to its good balance between security, key size, and computational efficiency. The security of lattice cryptography is based on lattice hard problems, such as Ring Learning With Errors (RLWE) and its generalized form, Module-LWE. The NIST-standardized ML-KEM (Kyber) and ML-DSA (Dilithium) are both based on the Module-LWE problem. Figure 1 categorises the five main families of post-quantum algorithms and indicates their status within the NIST standardisation process. HQC was selected by NIST in Round 4 for standardization, while Classic McEliece and BIKE were not selected; Rainbow and SIKE were broken and are no longer under consideration [4]. In addition, NIST requires the federated system to complete the migration by 2035.

Table 1 compares the suitability of major PQC algorithm families for embedded scenarios [14].

### 2.3. Resource-Constrained Characteristics of Edge Devices

Edge devices are computing nodes deployed at the network edge, close to data sources; examples include sensors, smart cameras, industrial controllers, and wearable devices. According to the classification in RFC 7228, IoT devices fall into three categories [15], as detailed in Table 2.

A vast number of industrial edge nodes fall into the Class-0 or Class-1 categories; compared to cloud servers, they exhibit the following typical resource constraints:

1. Limited computing power: Clock speeds typically range from tens to hundreds of MHz, and they lack floating-point units (FPUs) or dedicated cryptographic instruction sets. For instance, the ARM Cortex-M0+ implements only the ARMv6-M instruction set (comprising approximately 52 instructions) and lacks 64-bit multiplication capabilities as well as DSP and SIMD extensions [1].
2. Scarce storage resources: RAM capacity typically ranges from a few kilobytes to a few hundred kilobytes, and Flash storage is also severely limited.
3. Tight energy budgets: Many devices rely on battery power, making energy consumption a primary design constraint.
4. Limited communication bandwidth: Low-power wireless protocols (such as ZigBee, LoRa, and BLE) are commonly used, resulting in low transmission rates and small packet sizes.

These constraints pose significant challenges for the direct deployment of standard PQC algorithms on edge devices. Taking ML-KEM-512 as an example, a typical implementation on the ARM Cortex-M4 platform requires approximately 10 KB of RAM and millions of clock cycles; for a Class-1 sensor node equipped with only 16 KB of RAM, this imposes a substantial resource burden.

## 3. Challenges in Deploying Post-Quantum Technologies on Edge Devices

### 3.1. Device Heterogeneity and Physical Integration Difficulties

Deploying QKD systems requires specialized hardware, including quantum light sources, single-photon detectors, quantum random number generators, and associated optical and electronic systems. A standard QKD setup typically occupies several rack units (RU) and consumes power ranging from tens to hundreds of watts [16]. These characteristics conflict with the design goals of miniaturization, low power consumption, and low cost for edge nodes.

Specifically, QKD equipment faces the following physical integration obstacles in edge scenarios:

1. Physical Form Factor Mismatch. Industrial sensor nodes are typically designed as compact embedded modules with dimensions on the centimeter scale. In contrast, QKD equipment, which comprises optical platforms, temperature control systems, and precision circuitry, is difficult to integrate into such compact form factors.
2. Difficulty in meeting power budgets. Typical edge nodes operate within a power budget ranging from milliwatts to a few watts. QKD equipment, however, typically consumes 10 to over 100 times that amount of power, making it difficult to operate using batteries or energy-harvesting devices. Furthermore, QKD hardware components, such as quantum random number generators and single-photon detectors, incur higher costs [17].
3. Insufficient environmental adaptability. Industrial environments involve uncertainties such as vibration, temperature fluctuations, and electromagnetic interference, whereas QKD systems demand precise optical alignment and environmental stability. Consequently, the commercialization of QKD in edge scenarios remains challenging, as high module costs hinder both service providers and end-users from achieving a break-even point.

In contrast, PQC schemes, being purely software-based algorithms, possess inherent advantages regarding physical integration, as they simply require software execution on existing embedded processors. However, PQC schemes also face significant challenges concerning computational and storage resource requirements; details are provided in Section 3.2.

### 3.2. Constraints on Computational and Storage Resources

Compared to traditional public-key cryptography (RSA/ECC), PQC algorithms generally exhibit significantly higher resource requirements. Table 3 compares the resource requirements of representative PQC schemes and traditional schemes on embedded platforms; the data is based on the latest benchmarking studies [1].

As shown in Table 3, the resource requirements of PQC schemes are generally several times to an order of magnitude higher than those of traditional schemes. Of particular note:

ML-DSA signature operations exhibit high latency variability; due to the rejection sampling mechanism, the coefficient of variation reaches 66–73%, and the 99th-percentile latency for ML-DSA-87 can reach 1125 ms. This imposes significant performance constraints on industrial control scenarios with strict real-time requirements.

The memory requirements of ML-DSA increase rapidly with the security level: ML-DSA-44 requires 35 KB of RAM, ML-DSA-65 requires 56 KB, and ML-DSA-87 requires 91 KB. This makes it difficult for Class-0 devices (with approximately 10 KB of RAM) to run any ML-DSA scheme.

Crucially, the peak memory footprint of PQC operations often significantly exceeds their average consumption. Taking the ML-DSA signature generation process as an example, the iterative nature of the algorithm requires the system to maintain large data structures in memory, leading to a rapid increase in stack space requirements. This issue is particularly acute for microcontrollers lacking a Memory Management Unit (MMU), where a stack overflow directly results in a system crash. Table 4 and Table 5 compare the resource requirements of PQC algorithms versus classical algorithms on embedded platforms; these requirements are normalized against ECC-256.

Table 4 and Table 5 compare classical algorithms, mainstream PQC schemes, and the proposed scheme in terms of memory usage and time overhead. The tables clearly show that PQC schemes (particularly ML-DSA) impose resource demands exceeding the capabilities of Class-0 and Class-1 edge devices; in contrast, the proposed hierarchical offloading strategy brings the overhead of edge nodes back to a level comparable to that of traditional schemes.

For instance, ML-KEM-512 requires approximately 20–30 KB of RAM for key generation and encapsulation. While benchmark tests on Cortex-M0+ platforms demonstrate that executing this algorithm is technically feasible, it poses severe system-level challenges for typical Class-1 nodes with only 16 KB of RAM. The memory requirement inherently exceeds the total available capacity, making standalone execution impossible without external memory. Furthermore, even if sufficient RAM were available, allocating such a large contiguous block would consume the majority of the device’s memory, leaving insufficient space for application logic and network stacks, thereby risking system instability.

### 3.3. Protocol Compatibility Gap

Industrial IoT systems make extensive use of established communication protocols such as Modbus, Profinet, CANopen, and MQTT [18]. These protocols were not designed with quantum security requirements in mind; their data frame structures, session management mechanisms, and key exchange processes are all built upon classical cryptographic systems.

Integrating QKD or PQC into existing industrial protocol stacks presents the following compatibility challenges:

1. Frame structure incompatibility. Data frames in traditional industrial protocols typically follow a fixed format with limited space reserved for key material. In contrast, the sizes of PQC public keys and ciphertexts (ranging from hundreds of bytes to several kilobytes) far exceed the space allocated by traditional schemes. Forcing an expansion of the frame structure would compromise protocol compatibility and necessitate firmware modifications across network nodes.
2. Differences in session mechanisms. QKD key distribution operates as an independent physical-layer process, maintaining a high degree of decoupling from upper-layer protocols. Conversely, PQC Key Encapsulation Mechanisms (KEMs) must be embedded within session establishment workflows such as TLS or IPsec. Seamlessly integrating KEM processes into industrial protocols while ensuring backward compatibility with existing equipment remains a critical technical challenge.
3. Conflict regarding real-time requirements. Industrial control scenarios impose strict constraints on latency (typically requiring millisecond-level response times). The additional computational latency introduced by PQC, ranging from a few milliseconds to tens of milliseconds or even seconds, can exceed the time budget of the control cycle.

Research indicates that protocols such as Modbus TCP/IP lack built-in security mechanisms (e.g., authentication, integrity verification, and encryption). Researchers have proposed proxy-based methods that utilize PQC (specifically Kyber and Dilithium) to secure legacy PLCs without modifying their firmware [19]. This serves as a crucial technical foundation for the middleware solution presented in Section 4.2 of this work.

### 3.4. Engineering Cost Pressures of Large-Scale Deployment

The large-scale deployment of quantum-resistant security involves not only technical issues but also challenges related to systems engineering and economics:

(1) Equipment replacement costs. For the existing installed base of edge devices (numbering in the billions globally), fully replacing them with new equipment that supports QKD or PQC would incur massive hardware costs. Estimates suggest that the deployment cost per node could be several times higher than that of a pure QKD solution [19].
(2) Increased operational complexity. QKD systems require periodic calibration, and key management involves complex lifecycle processes. In large-scale distributed edge networks, the generation, distribution, updating, and revocation of keys would impose a significant operational burden.
(3) Risks associated with technology selection. Although PQC standards have been released, the long-term security of the algorithms still requires time to be verified. Committing prematurely to a specific technological path could lead to compatibility risks arising from the future evolution of standards.
(4) Talent and knowledge gaps. Quantum-resistant cryptography involves interdisciplinary knowledge spanning quantum physics, mathematics, cryptography, and embedded systems. Industrial enterprises generally lack technical teams possessing such multidisciplinary expertise.

## 4. Lightweighting Strategies

To address the four key challenges identified in Section 3, this section proposes three synergistic lightweighting strategies.

### 4.1. Hierarchical Quantum-Safe Deployment Architecture Addressing Device Heterogeneity

**Figure 2:**
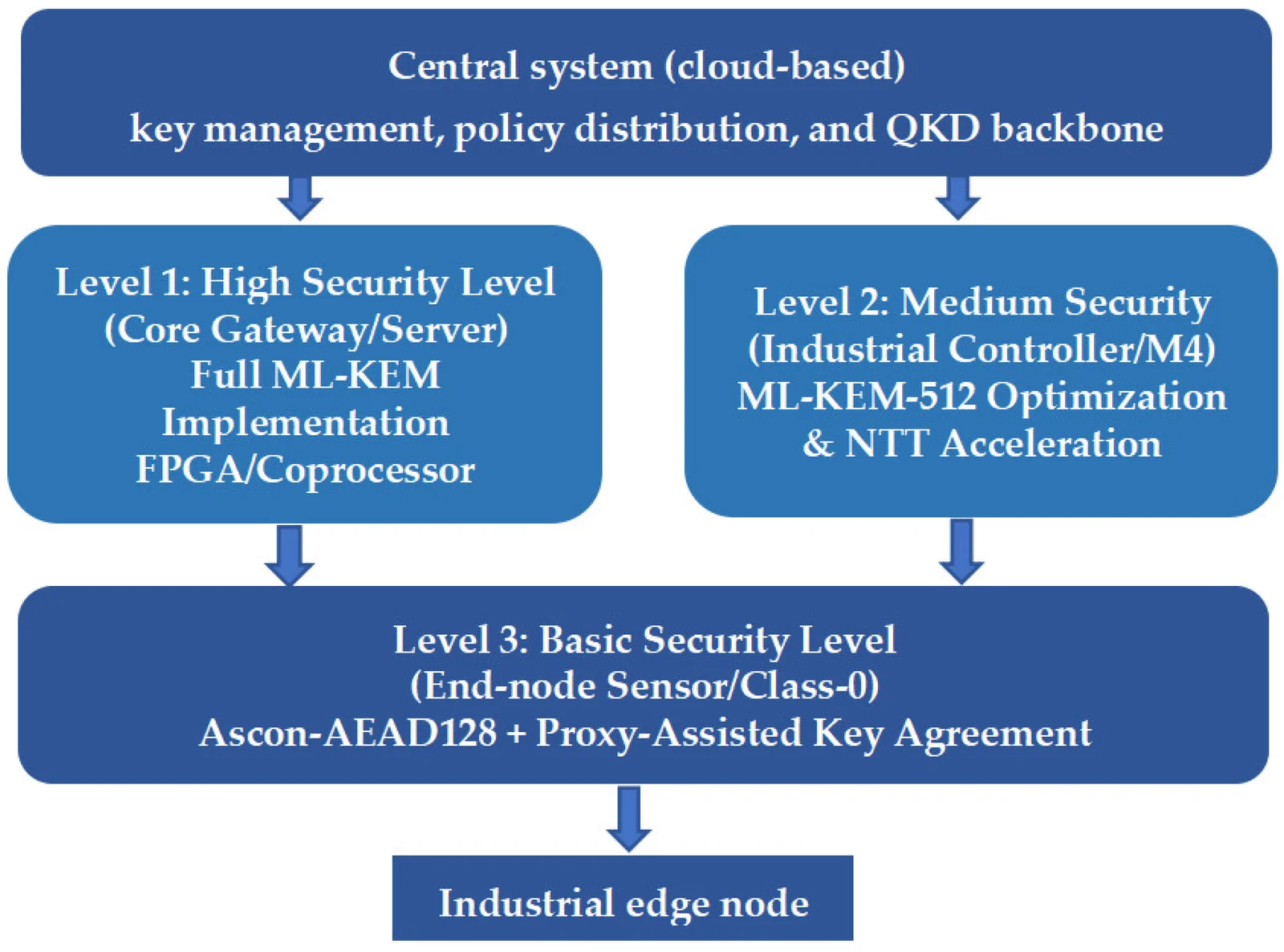
Hierarchical deployment architecture for quantum security addressing device heterogeneity.

Not all edge devices require the same level of quantum-resistant protection. This work proposes a hierarchical deployment architecture, specifically a quantum-safe hierarchical deployment architecture addressing device heterogeneity (Figure 2), that categorizes edge devices into three security levels. It is important to distinguish this security Level from the device capability Class defined in RFC 7228. Our architecture assigns a security Level to devices based on their Class.

Level 1 (High Security): Deployed on nodes with robust computing and storage capabilities, such as core gateways and edge servers. These devices are equipped with dedicated cryptographic coprocessors or FPGA accelerators to fully implement the standard ML-KEM and ML-DSA algorithms. This level is suitable for core nodes handling sensitive data (e.g., payment information, identity authentication credentials). Research by Sadeghi et al. demonstrated that a hardware-accelerated ML-KEM implementation achieves significant performance gains (twice as fast as a software implementation) in an SoC-level design [20].

Level 2 (Medium Security): Deployed on industrial controllers and smart sensors with moderate resources (e.g., ARM Cortex-M4/M7, >64 KB RAM). It utilizes lightweight parameter sets (such as ML-KEM-512) or optimized PQC implementations, reducing resource overhead through algorithmic optimizations (e.g., NTT acceleration, pre-computation). The optimized implementation by Botros et al. more than doubled NTT speeds [7].

Level 3 (Basic Security): Deployed on resource-constrained end sensors (e.g., Class-0 devices with only a few KB of RAM). In August 2025, NIST officially released the lightweight cryptography standard SP 800-232, based on the Ascon algorithm family [21]. The scheme proposed in this work adopts Ascon-AEAD128 for symmetric encryption at the basic security level, relying on higher-level nodes to perform PQC computations only for critical operations such as key exchange [21]. Ascon’s design prioritizes resistance to side-channel attacks, a feature particularly crucial for small, resource-constrained embedded devices.

The core concept of this tiered architecture is the “dynamic trade-off between security strength and resource overhead.” Specifically for Class 0/1 devices, while running lightweight PQC algorithms like ML-KEM-512 is theoretically possible, it is systemically inefficient. Therefore, our architecture offloads these intensive asymmetric operations to a more capable proxy. This allows the constrained devices to rely on lightweight symmetric cryptography (e.g., Ascon) for daily communications, preserving their scarce RAM and energy for primary sensing and control tasks.

Security considerations for key storage and root of trust: While the tiered architecture primarily focuses on computational offloading, secure key storage is equally critical. For Level 1 and Level 2 devices equipped with hardware security features, we recommend the following:

Secure key storage: Private keys and long-term secrets should be stored in a secure element (e.g., ATECC608A) or in the device’s Trusted Execution Environment (TEE) where available. For devices without dedicated secure storage, keys should be encrypted under a device-unique key derived from a physically unclonable function (PUF) or from a key diversified from a master key stored in the proxy [20].

Hardware root of trust: Level 1 devices (core gateways) should incorporate a hardware root of trust, such as a dedicated cryptographic coprocessor or FPGA-based PQC accelerator [22,23], to ensure that PQC operations are executed in a tamper-resistant environment. This prevents an attacker with physical access from extracting secret key material during computation.

For Class 0/1 devices without secure elements: These devices store only ephemeral session keys (derived from the proxy-assisted key agreement) in plaintext in SRAM, which is cleared upon reboot. Long-term secrets are not stored on these devices; instead, they are re-provisioned by the proxy at each boot using the certificate-based authentication described in Section 4.3.1.

### 4.2. Middleware for Quantum-Classical Hybrid Network Protocol Coordination

**Figure 3:**
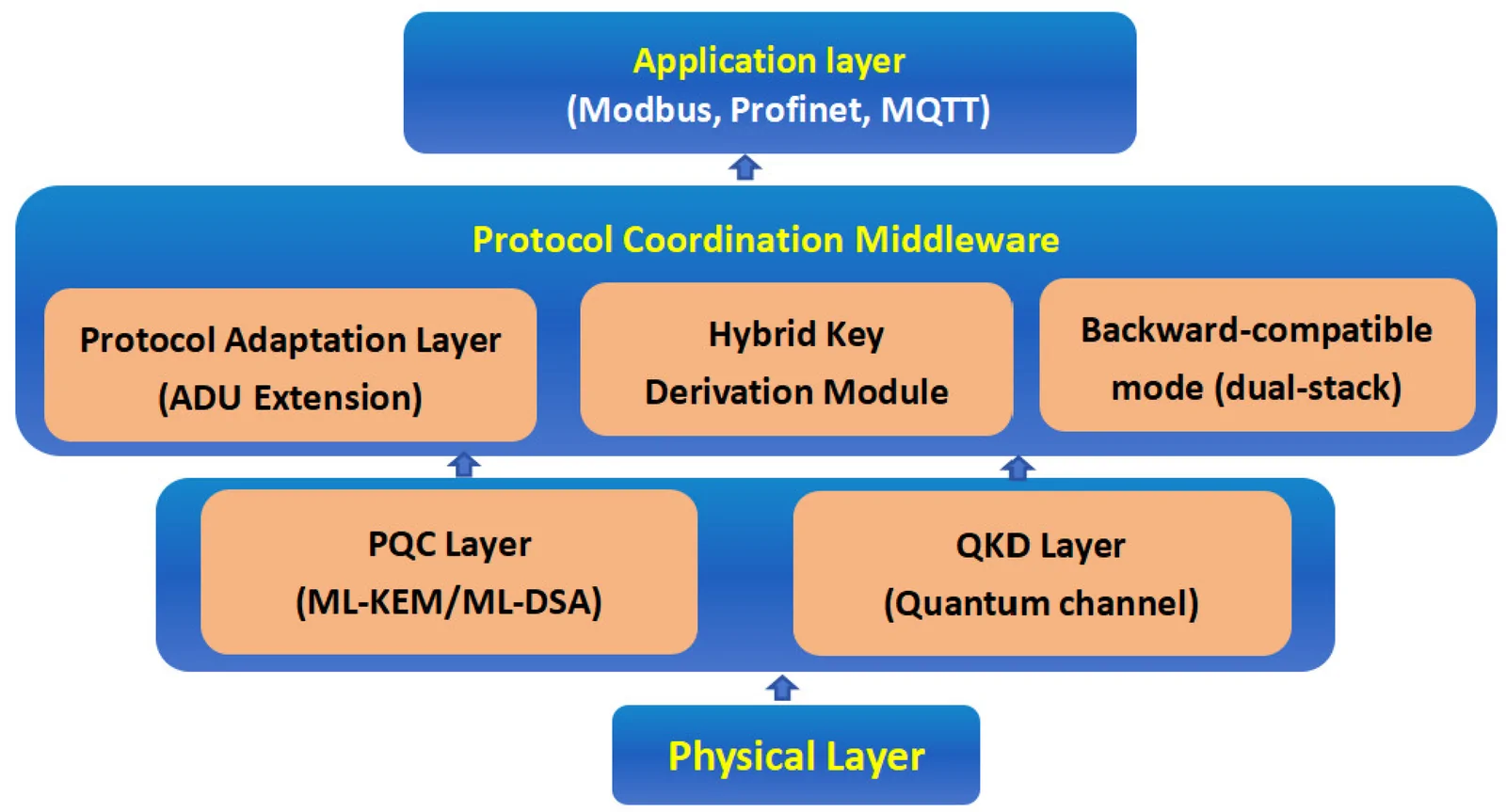
Architecture of the collaborative middleware for hybrid quantum-classical network protocols.

To bridge the compatibility gap between QKD/PQC and existing industrial protocols, this work proposes a middleware architecture for quantum-classical hybrid network protocol coordination, as shown in Figure 3. Consistent with the proxy-based legacy ICS security framework proposed by Trungadi et al. [23], this architecture sits between the quantum-resistant security layer and the existing industrial protocol layer, performing the following functions:

(1) Protocol adaptation and transparent conversion. The middleware automatically maps PQC key encapsulation/decapsulation processes to the key negotiation workflows of industrial protocols. For protocols that do not support dynamic key negotiation—such as Modbus—the middleware achieves a “transparent upgrade” by embedding key material within the Application Data Unit (ADU) at the application layer, without requiring modifications to the underlying protocol stack.
(2) Hybrid key derivation protocol. The middleware combines quantum keys distributed via QKD with post-quantum keys negotiated via PQC through the following procedure:

Let *K_QKD_* be the key material obtained from the QKD backbone (256 bits), and let *K_PQC_* be the shared secret from ML-KEM-512 encapsulation (shared secret of 32 bytes). The final session key *K_session_* is derived as:

*K_session_* = KDF(*K_QKD_* ‖ *K_PQC_* ‖ transcript ‖ nounce ‖ label)

where KDF is instantiated with SHA-256 (or HKDF-SHA-256), transcript is the concatenation of all handshake messages exchanged between the two parties (including identities, nonces, and algorithm identifiers), and label is a domain-separation string. The complete handshake proceeds as follows:

① Session initiation: The initiating party sends a *Hello* message containing its identity, supported PQC and QKD algorithm suites, and a fresh nonce.
② QKD key material: For links with QKD capability, the QKD system distributes KQKD to both endpoints through the quantum channel. The classical channel used for QKD sifting is authenticated using pre-shared keys or certificates provisioned by the central system.
③ PQC key exchange: The responding party generates an ML-KEM key pair, sends the public key to the initiator, who responds with the ciphertext. Both parties compute *K_PQC_*.
④ Key confirmation: Both parties compute *K_session_* independently and exchange a confirmation MAC over a known test string using *K_session_*.

Authentication of the classical channel in QKD: The classical channel used for basis reconciliation and error correction in QKD is authenticated using the same certificate-based infrastructure as the PQC layer. This prevents man-in-the-middle attacks on the QKD classical channel, which is a known vulnerability in QKD implementations.

(3) Backward-compatible mode. To address the engineering challenges associated with migrating to post-quantum cryptographic systems, the middleware layer employs a dual-stack parallel architecture. This architecture enables new terminals to utilize PQC/QKD protocols for key exchange while maintaining backward compatibility with legacy devices using traditional RSA/ECC protocols. Through this hybrid negotiation mechanism, the system achieves a seamless transition at the service level, significantly mitigating the risk of systemic paralysis caused by the mandatory replacement of underlying cryptographic algorithms upon the arrival of “Q-Day” [24].

### 4.3. Three-Tier Collaborative Key Management: “Edge Node-Network Proxy-Central System”

**Figure 4:**
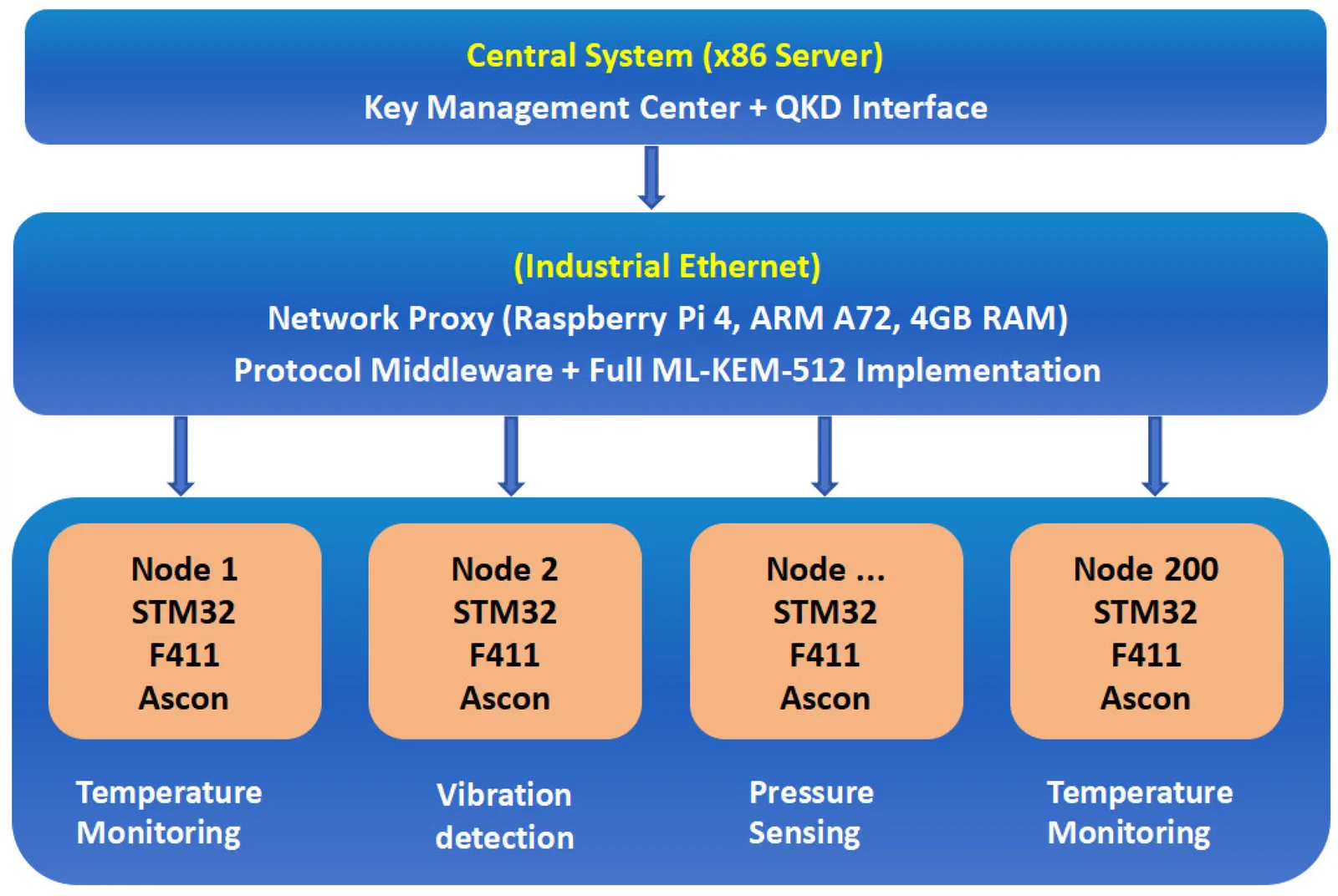
Three-tier collaborative key management architecture (“Edge Node-Network Proxy-Central System”).

To address the computational and storage bottlenecks of edge devices, this work proposes a distributed key management mechanism featuring a three-tier collaboration among edge nodes, network proxies, and the central system (see Figure 4). The three tiers are defined as follows, using descriptive names rather than numeric levels to avoid confusion with the security levels defined in Section 4.1.

Bottom Tier-Edge Node Layer: End sensors are responsible only for lightweight symmetric encryption (Ascon-AEAD128) and hash operations. Computationally intensive tasks, such as PQC public-key operations and signature verification, are offloaded to higher-level nodes. Edge nodes store only the short-term keys required for the current session (typically just a few bytes), thereby significantly reducing storage requirements.

Middle Tier-Network Proxy Layer: Deployed at edge gateways or fog nodes. Proxy nodes perform the following functions: (1) conducting PQC key exchange on behalf of the edge nodes they manage; (2) caching and managing key materials for subordinate nodes; and (3) relaying and distributing keys among nodes. Possessing superior computational capabilities (such as ARM Cortex-A series or x86 processors), proxy nodes are capable of handling full PQC operations [25].

Top Tier-Central System Layer: A cloud-based key management center. It is responsible for: (1) formulating and disseminating global key policies; (2) managing the QKD backbone network (e.g., for high-value links); (3) managing the full key lifecycle (generation, distribution, update, revocation, and archiving); and (4) detecting and responding to anomalous behavior.

The core innovation of this three-tier mechanism lies in “shifting”, rather than replicating, computationally and storage-intensive operations to high-resource nodes; edge nodes do not need the capability to execute PQC algorithms themselves, but can instead obtain quantum-resistant keys simply by interacting securely with proxy nodes via lightweight protocols [26].

#### 4.3.1. Trust Establishment and Security Assumptions

The initial trust relationship between an edge node and its network proxy is established through a secure bootstrap procedure. Each edge node is pre-provisioned at manufacturing time with a unique device identity (DevID) and a corresponding pre-shared key (PSK) or certificate signed by the central system’s root of trust. During the first connection, the edge node authenticates the proxy by verifying its certificate against the root of trust stored in the device’s secure element (if available) or in protected flash memory. The proxy similarly authenticates the edge node using the DevID and a challenge-response protocol based on the pre-provisioned key material.

The proxy is authenticated at each session renewal using the same certificate-based mechanism, with certificate revocation lists (CRLs) periodically distributed by the central system to all proxies.

Security guarantees under proxy compromise: If a proxy node is compromised, the adversary gains control over the proxy’s key material and can intercept communications between the proxy and its managed edge nodes. However, the following protections remain:

(1) End-to-end encryption between edge nodes and the central system: For critical data flows, edge nodes can establish direct secure channels with the central system using pre-provisioned keys, bypassing the proxy for data confidentiality (though key agreement still relies on the proxy for PQC operations).
(2) Forward secrecy: Session keys derived through the hybrid QKD/PQC mechanism are ephemeral and independent. Compromise of a proxy does not reveal past session keys, as these are derived from fresh PQC key exchanges and are not stored persistently.
(3) Replay resistance: Each key exchange includes a fresh nonce and timestamp, bound into the KDF inputs. Replayed messages are detected by the receiving party’s nonce cache.
(4) Downgrade resistance: The protocol negotiates the highest available security level supported by both parties. Any attempt to downgrade to a weaker algorithm is detectable because the security level identifier is included in the authenticated transcript.
(5) Man-in-the-middle resistance: All protocol messages are authenticated using the edge node’s DevID and the proxy’s certificate. The full transcript is bound into the key derivation process, preventing MITM attacks that alter protocol messages.

#### 4.3.2. Symmetric Key Provisioning for Edge Nodes

After the proxy completes the PQC key exchange with the central system (or with another proxy), it derives a unique session key for each managed edge node using the following procedure:

(1) The proxy generates a fresh 128-bit symmetric key *K_node_* using a cryptographically secure random number generator.
(2) *K_node_* is encrypted under the hybrid session key *K_session_* (derived from QKD/PQC as described in Section 4.2) and transmitted to the edge node.
(3) The edge node decrypts Knode using its pre-provisioned key material (or using a key derived from the initial bootstrap procedure) and stores it in volatile memory for the duration of the session.
(4) *K_node_* is used for all subsequent symmetric encryption operations (Ascon-AEAD128) between the edge node and its proxy until the session expires or is explicitly renewed.

This approach ensures that (1) edge nodes never handle PQC keys directly, (2) each node has a unique symmetric key, limiting the impact of a single node compromise, and (3) key renewal can be performed independently per node without affecting others.

Security of edge-proxy communication: All communications between edge nodes and their network proxies are protected using the following mechanisms:

(1) Confidentiality: All data exchanged between the edge node and the proxy is encrypted using Ascon-AEAD128 with the per-node session key *K_node_*.
(2) Integrity and authenticity: Each message includes a 128-bit authentication tag generated by Ascon-AEAD128, providing integrity protection and source authentication.
(3) Replay protection: Each encrypted message includes a monotonically increasing sequence number and a timestamp, verified by the receiving party. Out-of-order or replayed messages are rejected.
(4) Freshness: Each session key *K_node_* has a configurable lifetime (default: 30 min in our experiment). Upon expiration, the proxy initiates a fresh key distribution using the procedure described above, ensuring that a compromised key does not remain valid indefinitely.
(5) Physical security assumptions: For Class 0/1 devices without tamper-resistant hardware, we assume that physical attacks (e.g., side-channel or probing) are outside the current threat model. However, the short key lifetime limits the window of opportunity for such attacks.

## 5. Experimental Verification and Case Analysis

### 5.1. Experimental Scenarios and Configuration

**Figure 5:**
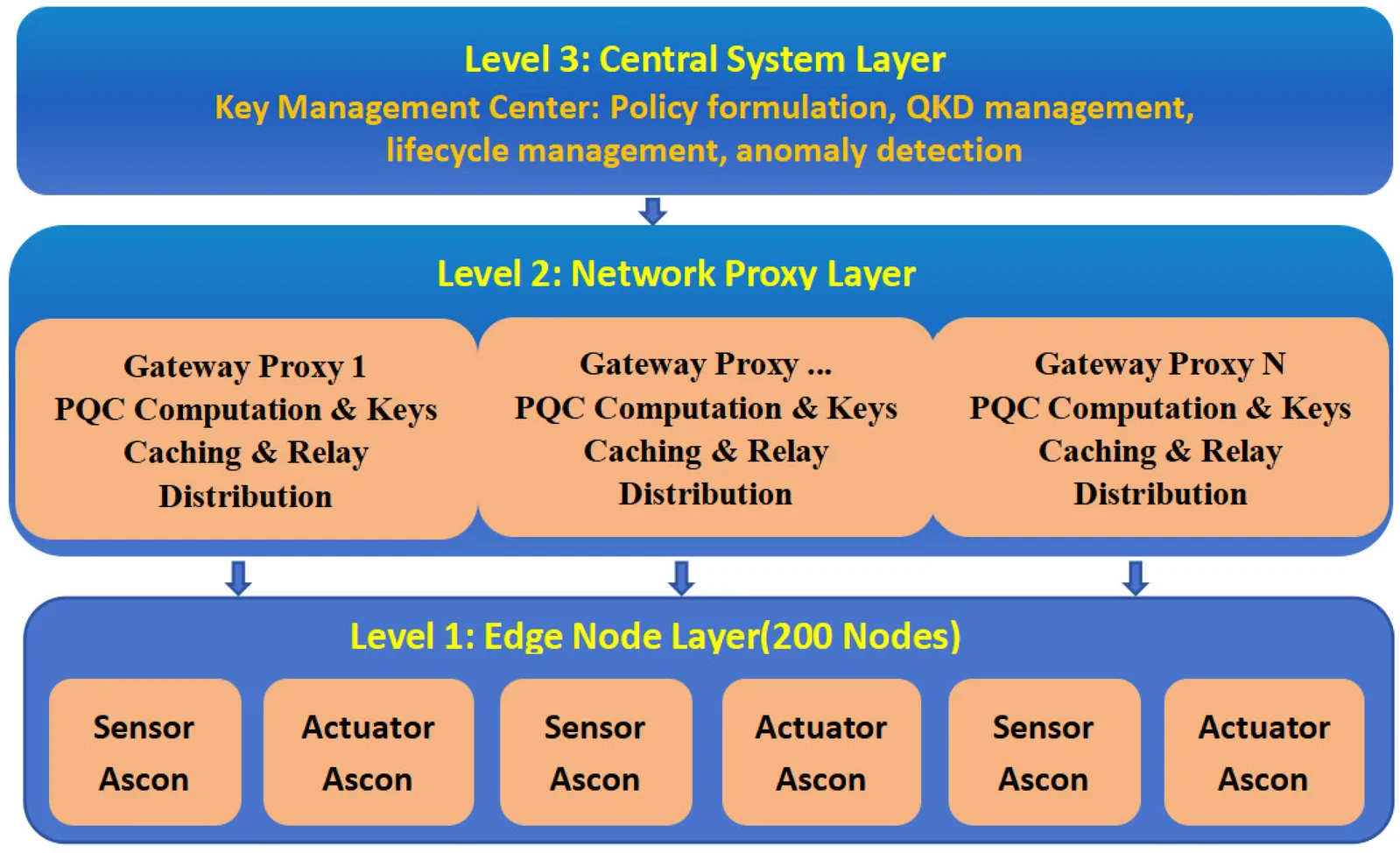
Hardware configuration of the experimental platform.

To verify the effectiveness of the lightweight countermeasures proposed in this work, we conducted experiments within the sensor network of a smart manufacturing plant. The plant utilizes approximately 200 sensor nodes deployed for applications such as temperature monitoring, vibration detection, and pressure sensing on production lines. The experimental configuration is detailed in Table 6, and the hardware setup of the experimental platform is shown in Figure 5.

The edge node is implemented on an STM32F411CEU6 microcontroller (Cortex-M4, 128 KB RAM). Note on Experimental Setup and Table 3: The selection of this Class 2 device is intended to quantitatively validate the performance gains of our computation offloading mechanism. As shown in Table 3, while ML-KEM-512 can execute on a lower-tier Cortex-M0+ core, the resulting memory footprint severely limits its practical deployment in Class 0/1 environments. By demonstrating a ~40-fold reduction in computational overhead on the STM32F411, we empirically prove that offloading is highly effective. For true Class 0/1 devices, our architecture is not merely an optimization but a prerequisite for functional feasibility, as it prevents the memory exhaustion that would occur if they attempted to run PQC independently.

#### Experimental Methodology Details

The proxy software consists of two components: (1) the protocol coordination middleware, version 1.0.0, implemented as a user-space daemon; and (2) the PQClean library, commit 3730b32a, providing the ML-KEM-512 implementation. Edge node firmware was compiled with ARM GCC 10.3.1 using -Os (optimize for size) with *-mthumb -mcpu=cortex-m4 -mfpu=fpv4-sp-d16 -mfloat-abi=hard*. Proxy software was compiled with GCC 11.2.0 using *-O3 -march=armv8-a+crc*.

Measurement methodology: Latency was measured using the DWT cycle counter on Cortex-M4 (calibrated against a 100 MHz clock) and *clock_gettime(CLOCK_MONOTONIC)* on the proxy. Energy consumption was measured using a Nordic Power Profiler Kit II sampling at 100 kHz. All measurements were repeated 1000 times per operation; reported values are arithmetic means with 95% confidence intervals < ±3% of the mean.

Middleware implementation: The protocol coordination middleware was implemented as a user-space daemon on the proxy, intercepting Modbus TCP packets on port 502 and MQTT packets on port 1883. Key material was embedded in the Modbus Application Data Unit (ADU) as a vendor-specific extension. For MQTT, key material was carried in the user properties field of CONNECT and PUBLISH packets.

Frame format: For Modbus TCP, the key material extension uses the following format: *[0xFE][key_length (2 bytes)][key material][MAC (16 bytes)]* inserted after the standard MBAP header.

Supplementary experiment on Cortex-M0+: To validate the proposed architecture on genuinely constrained devices (Class 0/1), we additionally deployed the lightweight client on an RP2040 microcontroller (Cortex-M0+ dual-core, 133 MHz, 264 KB SRAM). The client executes only Ascon-AEAD128 encryption and lightweight protocol interactions, with all PQC operations offloaded to the proxy. PQC operations on the proxy used the PQClean library (commit 3730b32a) with the ML-KEM-512 implementation [27]. Measurements show:

- Peak stack usage: 3.2 KB (vs. 9.4 KB for native ML-KEM-512 decapsulation on the same platform)
- Total RAM footprint: 6.8 KB (vs. 12.1 KB for native ML-KEM-512)
- Energy per encryption operation: 0.08 mJ (vs. 2.83 mJ for a full ML-KEM-512 handshake)

These results confirm that the proxy-offloading architecture makes quantum-resistant security feasible even on Class 0/1 devices that lack sufficient resources to execute PQC algorithms natively.

### 5.2. Performance Test Results

The performance evaluation covers three dimensions: the computational overhead incurred at the edge node, the end-to-end key establishment performance of the proxy-mediated pipeline, and the communication and key-management overhead of the overall system.

(1) Computational performance

Based on the tiered deployment architecture, the system offloads computationally intensive NTT operations and polynomial multiplications to proxy nodes, allowing edge nodes to handle only lightweight Ascon-AEAD128 encryption and basic protocol interactions. As reported in the upper panel of Table 7, this mechanism reduces the computational overhead on edge nodes by a factor of approximately 40 and cuts memory usage by about 60%, significantly enhancing operational efficiency at the edge.

While the upper panel of Table 7 quantifies the reduction in edge-node computational burden, it is equally important to characterize the complete key establishment pipeline from a system perspective. Accordingly, the lower panel of Table 7 summarizes the end-to-end performance metrics, including proxy computation time, communication latency, system throughput, and scalability under concurrent requests. The proxy completes ML-KEM-512 key generation and encapsulation in 18.6 ms on the Cortex-A72 platform, and the total end-to-end key establishment latency averages 25–30 ms, which satisfies the sub-200 ms real-time requirements of typical industrial control scenarios. With 10 proxy nodes operating in parallel, the system can handle approximately 350 concurrent key agreement requests per second, demonstrating adequate scalability for the 200-node deployment.

(2) Communication overhead

By optimizing the frame structure through the protocol coordination middleware, the communication overhead of a single key exchange is significantly reduced; specific data are presented in Table 8. This mechanism holds significant application value for low-bandwidth industrial wireless networks such as LoRa and ZigBee. The middleware achieves lossless compatibility with the existing Modbus TCP frame structure by embedding streamlined key material into the Application Data Unit (ADU).

(3) Key management efficiency

The three-level collaborative key management mechanism enables on-demand key distribution and automatic updates. Over a 30-day experimental period across the 200-node deployment, the system executed a total of 1247 key negotiation and update operations at the network proxy level (i.e., aggregated across all proxies, representing group-key renewals and individual node key refreshes). Key performance indicators are summarized in Table 9. Test results indicate an average key negotiation latency of 42 ms and a maximum latency of 187 ms, satisfying the real-time requirements, typically under 200 ms, of industrial control scenarios. Furthermore, to address the high latency variability associated with ML-DSA signing operations (with a coefficient of variation ranging from 66% to 73%), the proposed scheme offloads signing tasks to proxy nodes, effectively bypassing this bottleneck and ensuring that edge nodes are not required to perform ML-DSA signing directly.

### 5.3. Deployment Cost Analysis

Currently, the average construction cost for a QKD system is approximately RMB 7 million per kilometer, with an annual operating cost of about RMB 1.4 million [28]. The specific cost breakdown and estimates are as follows:

(1) Hardware Equipment (accounting for~60% of total costs)

This constitutes the core of the cost and primarily includes the following key modules:

QKD terminal equipment: This is the system’s core component; currently, the cost of a single fiber-based QKD node ranges from RMB 1 million to RMB 3.5 million. Fortunately, prices are dropping as technology advances; in the future, the shift toward chip-based integration is expected to reduce the price of single-channel modules to below RMB 350,000.

Core components: The high cost of the equipment stems from the high-end components within it. For instance, single-photon detectors (SPADs), which serve as the system’s “heart”, are expensive. Quantum random number generators (QRNGs), used to produce true random numbers, also require specialized chips.

High-precision environmental control: QKD equipment is extremely sensitive to its operating environment; precise temperature control and anti-vibration systems are required to ensure the stability of photon polarization states, which also adds to the cost.

(2) Network Construction (~25% of total cost)

This component primarily relates to fiber-optic infrastructure.

Fiber-optic laying/leasing: This is one of the major variable costs.

Traditional approach (dedicated dark fiber): QKD signals are extremely weak, akin to “fireflies”, and typically require exclusive use of a fiber strand (“dark fiber”), as they cannot be mixed with high-power optical communication signals. In urban areas, the annual cost of leasing such a dedicated fiber pair can range from RMB 70,000 to RMB 350,000 per kilometer.

Emerging approach (co-propagation): Solutions enabling QKD signals and classical optical communication signals to coexist on the same fiber, using different wavelengths via Wavelength Division Multiplexing (WDM) technology, have matured. This method can reduce deployment costs by up to 60% and represents a key pathway to cost reduction.

Repeater deployment: Long-distance transmission requires the deployment of trusted relays to boost signals, which also significantly increases network construction costs.

(3) Maintenance and Operations (~15% of total costs)

This component represents ongoing expenditure.

Power consumption: QKD equipment, particularly single-photon detectors that require cooling, consumes significant power.

Environmental control: Equipment must operate in high-standard server rooms with constant temperature and humidity; the associated precision air conditioning and monitoring systems incur continuous power and maintenance costs.

Specialized maintenance: Troubleshooting QKD systems is complex; factors such as fiber vibration or temperature fluctuations can cause error rates to spike, requiring professional intervention.

**Figure 6:**
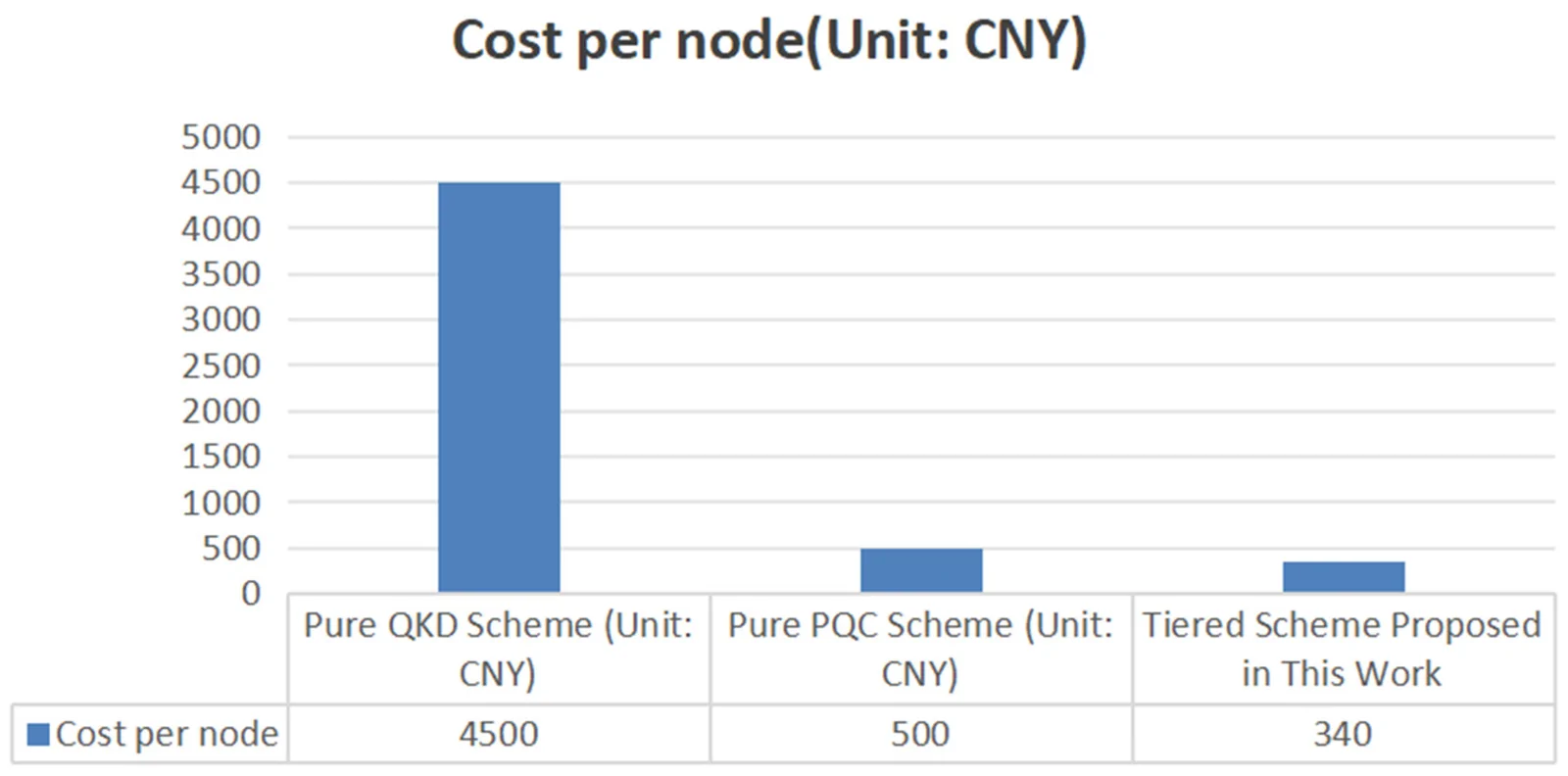
Comparison of single-node costs across three deployment schemes.

Table 10 compares the costs of the three deployment schemes at a scale of 200 nodes, and Figure 6 visualizes the per-node cost comparison across the three schemes. The pure QKD scheme incurs prohibitive costs, as it necessitates equipping each edge node with dedicated QKD hardware or establishing fiber optic links, rendering it economically unviable for large-scale edge deployment. While a pure PQC scheme eliminates QKD hardware expenses, it necessitates sufficient computational capability at edge nodes to execute PQC algorithms, resulting in significant hardware upgrade costs [29]. In contrast, the hierarchical scheme proposed in this work offloads PQC computations to proxy nodes, allowing edge nodes to rely solely on lightweight symmetric encryption algorithms (such as Ascon), thereby substantially reducing hardware upgrade costs.

Experimental results demonstrate that the proposed scheme ensures quantum-resistant security while reducing the single-node deployment cost by approximately 92.4% compared to a pure QKD scheme and by about 32% compared to a pure PQC scheme. Furthermore, the scheme achieved a seamless transition with zero service interruption, ensuring that the normal operation of the production line remained unaffected throughout the two-week phased deployment process.

### 5.4. Discussion and Limitations

Despite the positive experimental results, the proposed scheme has the following limitations:

(1) Proxy nodes act as single points of failure. At the network proxy layer, if a proxy node is attacked or fails, all edge nodes under its management lose access to post-quantum key services. The proxy single-point-of-failure risk is mitigated through two mechanisms: ① each edge node is configured with a prioritized list of backup proxies; ② the central system monitors proxy health via heartbeat messages and triggers automatic failover within <500 ms upon detecting a proxy failure [25]. In our 30-day experiment, no proxy failure occurred, but failover simulation tests showed a median switchover time of 320 ms, well within the industrial control tolerance of 500 ms. Future work could introduce redundancy mechanisms for proxy nodes and decentralized identity management.
(2) QKD integration is not yet fully mature. Current experiments primarily validate the PQC component, while QKD integration remains at the proof-of-concept stage. The deep hybridization of QKD and PQC, as well as their practical deployment performance in industrial environments, requires further validation.
(3) Long-term security remains to be seen. The security of PQC algorithms relies on mathematical problems that have not yet been fully broken. As cryptanalysis advances, certain parameter sets may require adjustment or upgrades.
(4) Validation on Extremely Constrained Devices. While the proposed architecture is theoretically applicable to Class 0 and Class 1 devices, this study’s experimental validation was conducted on a Class 2 device. Our earlier analysis (Section 3.2) and benchmarks (Table 3) confirm that while PQC algorithms can technically run on Cortex-M0+ cores, the associated memory and energy overheads are prohibitive for long-term deployment. Future work will involve implementing the ultra-lightweight client on microcontrollers with significantly fewer resources (e.g., ARM Cortex-M0+) to provide empirical data on the exact energy and latency savings achieved through our proxy-offloading strategy in the most constrained scenarios. Specifically, we plan to measure the reduction in peak current draw and the extension of battery life, which are the most critical metrics for Class 0/1 devices. Another critical security concern that requires further investigation is side-channel resilience.
(5) Side-channel security. The current implementation has not been fully evaluated for resilience against side-channel attacks (such as power analysis, timing attacks, and electromagnetic emissions). Although Ascon-AEAD128’s design prioritizes side-channel resistance [30] and the PQClean reference implementation of ML-KEM has undergone some side-channel evaluation [27], the PQC component on the proxy requires further hardening before deployment in high-security environments. Future work will involve:
  - Implementing constant-time NTT operations to prevent timing-based key extraction [30]
  - Evaluating power analysis resistance using test vector leakage assessment (TVLA) methodology [31]
  - Exploring masking schemes for ML-KEM on resource-constrained proxies [24,32]

For the edge nodes, which only execute Ascon-AEAD128, the side-channel attack surface is significantly smaller than for devices running full PQC algorithms. However, even lightweight implementations require careful attention to timing invariance.

## 6. Conclusions and Outlook

To address the four core challenges facing resource-constrained edge devices in the deployment of quantum-resistant security—namely device heterogeneity, resource limitations, protocol incompatibility, and high engineering costs—this work establishes a synergistic, lightweight mitigation framework. This framework comprises a tiered quantum-safe deployment architecture designed to resolve heterogeneity issues, a quantum-classical hybrid network middleware ensuring protocol compatibility, and an “Edge-Proxy-Center” three-tier distributed key management mechanism that supports large-scale deployment.

Experimental validation within a smart manufacturing factory’s sensor network demonstrates that the proposed scheme maintains post-quantum-resistant security capabilities (PQC component fully validated; QKD integration demonstrated at the proof-of-concept level) while reducing per-node deployment costs by approximately 92.4% compared to pure QKD solutions and by about 32% compared to pure PQC solutions; furthermore, it achieves a seamless transition with zero service interruption. The study confirms that lightweight algorithm design, hardware-software co-optimization, and tiered architectural deployment constitute three viable pathways for advancing IoT edge security into the quantum-resistant era.

Looking ahead, the following areas warrant further exploration:

(1) Adaptive security strategies. Researching adaptive mechanisms that dynamically adjust security levels based on device status, network environments, and threat intelligence is essential for achieving real-time optimization of both security and efficiency. Future research could explore the application of machine learning for dynamic parameter tuning [33].
(2) Hardware acceleration and novel architectures. With the rise of open instruction set architectures like RISC-V, designing dedicated instruction set extensions (such as NTT acceleration instructions) and lightweight hardware accelerators for PQC offers the potential for efficient quantum-resistant computation at extremely low power consumption. Unified instruction set extensions could support all NIST-approved PQC algorithms [34,35].
(3) Standardization and interoperability. Promoting the international standardization of lightweight quantum-resistant solutions is crucial to ensuring interoperability between devices from different vendors and mitigating the risk of ecosystem fragmentation [31].
(4) Side-channel security. Implementing PQC solutions that are resilient to side-channel attacks under resource-constrained conditions, thereby preventing key information leakage via physical means such as power analysis or electromagnetic emissions, is a key prerequisite for practical deployment [30,36].

The transition to quantum-resistant security is a long-term endeavor. For resource-constrained edge devices, there is no “one-size-fits-all” solution; only by flexibly combining various technical approaches, tailored to specific device capabilities, security requirements, and application scenarios, can we safeguard the security of the Internet of Things in the era of quantum computing.

## Figures and Tables

**Table 1:** Comparison of PQC algorithm families regarding suitability for embedded applications.

Algorithm Family	Representative Algorithms	Advantages	Embedded Challenges
Lattice-based cryptography	ML-KEM, ML-DSA	Moderate key sizes and balanced performance	Polynomial operation overhead, side-channel protection
Code-based cryptography	Classic-McEliece, HQC	High security margin	Large key size (>1 MB)
Hash-based cryptography	SPHINCS+/SLH-DSA	Simple primitive operations; secure and scalable	Large signature size, high computational cost
Multivariate cryptography	Rainbow, etc.	Compact keys in some schemes	Complex operations, insufficient maturity

**Table 2:** RFC 7228 Classification of IoT Devices.

Category	RAM (Data Memory)	Flash/ROM (Code Memory)	Brief Description
Class 0	≪10 KB	≪100 KB	Highly constrained; cannot directly run a full IP stack; relies on gateways or proxies for Internet communication; typical examples include passive RFID tags, temperature sensors (e.g., DS18B20 with minimal MCU), and simple contactless smart cards.
Class 1	~10 KB	~100 KB	Cannot run full HTTP/TLS protocol stacks; capable of running lightweight, constrained stacks (such as CoAP); does not require a gateway; can function as a peer node within an IP network. Typical examples include environmental sensors (temperature/humidity/pressure), motion detectors, and smart building actuators (e.g., STM32F1 series with 20 KB RAM).
Class 2	~50 KB	~250 KB	Less resource-constrained; in principle, capable of running full protocol stacks typical of laptops or servers, though lightweight protocols may still be chosen to reduce power consumption. Typical examples include industrial edge sensors with local processing capability, and IoT gateways for small subnets (e.g., STM32F4 series with 64–192 KB RAM).

Note: RFC7228 explicitly states that devices with capabilities significantly exceeding Class 2 are not further categorized; instead, they are treated simply as standard, unconstrained Internet devices.

**Table 3:** Comparison of resource requirements for representative cryptographic schemes on embedded platforms.

Scheme	Public Key Size (KB)	Private Key Size (KB)	Ciphertext/Signature Size (KB)	RAM Requirement (KB)	Execution Time (ms at 100 MHz)
ECC-256 (ECDH)	0.1	0.0	0.1	~2	~2.1
RSA-2048	0.3	1.2	0.3	~2	~10
ML-KEM-512	0.8	1.6	0.8	~20–30	~35.7
ML-KEM-768	1.2	2.4	1.2	~30–40	~50
ML-KEM-1024	1.6	3.2	1.6	~40–50	~70
ML-DSA-44	1.3	2.6	2.4	35	Signature: ~200
ML-DSA-65	2.0	4.0	3.3	56	Signature: ~400
ML-DSA-87	2.6	4.9	4.6	91	Signature: ~800

Note: ML-KEM data is based on actual measurements using an ARM Cortex-M0+ at 133 MHz; ML-DSA data is based on the Cortex-M4 [1]. It is worth noting that while ML-KEM-512 is executable on the Cortex-M0+, its RAM requirement (~20–30 KB) exceeds the total memory of a typical Class 1 device (e.g., 16 KB), rendering standalone operation infeasible without external memory. This underscores the necessity of our proposed offloading architecture for such constrained devices.

**Table 4:** Comparison of memory requirements between classical algorithms and PQC algorithms.

No.	Algorithm Name	Operation Type	Min RAM Usage (KB)	Max RAM Usage (KB)	Typical Value (KB)
1	ECC-256	Key exchange	1	2	1.5
2	RSA-2048	Encryption	1	2	1.5
3	ML-KEM-512	Key encapsulation	20	30	25
4	ML-KEM-1024	Key encapsulation	40	50	45
5	ML-DSA-44	Signature generation	35	35	35
6	ML-DSA-65	Signature generation	55	65	60
7	ML-DSA-87	Signature generation	91	91	91
8	This work (edge node)	Ascon-128 symmetric encryption+ protocol interaction	7	9	8

**Table 5:** Comparison of time performance between classical algorithms and PQC algorithms.

No.	Algorithm Name	Operation Type	Average Latency (ms)	P99 Latency (ms)
1	Ascon-128	Symmetric encryption	1.2	—
2	ECC-256	Key agreement	2.1	—
3	RSA-2048	Encryption	10	—
4	ML-KEM-512	Key encapsulation	35.7	—
5	ML-KEM-768	Key encapsulation	80	—
6	ML-KEM-1024	Key encapsulation	250	—
7	ML-DSA-44	Signature generation	~200	~600
8	ML-DSA-65	Signature generation	~400	~1500
9	ML-DSA-87	Signature generation	~800	~1125

**Table 6:** Experimental configuration for the smart manufacturing plant.

Item	Configuration
Edge Node (Level 3)	STM32F411CEU6 (ARM Cortex-M4, 100 MHz, 512 KB Flash, 128 KB RAM), Run a lightweight client (Ascon-AEAD128 + lightweight protocol interaction)
Network Proxy (Level 2)	Raspberry Pi 4 (ARM Cortex-A72, 1.5 GHz, 4 GB RAM), Run the protocol coordination middleware and the complete ML-KEM-512 implementation.
Central System (Level 1)	x86 server (Intel Xeon) running the Key Management Center and QKD interface.
Network Environment	Industrial Ethernet within the factory, utilizing a mix of Modbus TCP and MQTT protocols.
PQC Scheme	ML-KEM-512 (NIST Security Level 1)

**Table 7:** Computational overhead and end-to-end key establishment performance: native PQC execution on the edge node versus the proposed proxy-assisted lightweight scheme.

Category	Metric	Native ML-KEM-512 (on Edge)	Proposed Scheme (Proxy-Assisted)	Improvement
Edge-node overhead	Time per encryption/ encapsulation operation	50–80 ms @ 100 MHz	~1.2 ms (Ascon-AEAD128)	~40×
	RAM usage	20–30 KB	~8 KB	~60%
	Flash usage	~40 KB	~12 KB	~70%
	Energy consumption per operation	~4.0 mJ	~0.1 mJ	~40×
End-to-end key establishment (proxy-mediated)	Proxy PQC computation time (ML-KEM-512 keygen + encapsulation)	—	18.6 ms (Cortex-A72 @ 1.5 GHz, measured)	—
	Edge–proxy communication latency (round-trip, industrial Ethernet)	—	2–5 ms	—
	Total end-to-end key establishment latency	—	~25–30 ms	—
	System throughput (key agreements/s, single proxy)	—	~35 requests/s	—
	Scalability (concurrent requests, 10 proxies)	—	~350 requests/s	—

Note: The comparison reflects workload offloading rather than a cryptographic acceleration. The edge node no longer executes PQC operations; instead, it performs lightweight symmetric encryption, while PQC computations are delegated to the proxy. The 40× improvement represents the reduction in edge-node computational burden, not an acceleration of the PQC algorithm itself. Although the edge node does not perform PQC directly, the overall system remains quantum-resistant because the session keys are derived from the hybrid QKD/PQC mechanism at the proxy and the edge–proxy channel is secured by freshly generated symmetric keys, ensuring that the entire communication chain retains quantum-resistant security.

**Table 8:** Comparison of communication overhead.

Metric	Pure PQC Solution	Proposed Solution (Middleware Optimization)	Reduce the Proportion
Communication volume per negotiation	~2.4 KB	~0.8 KB	67%
Number of handshake round-trips	4×	2×	50%
Protocol frame modification	Requires	Not required	—

**Table 9:** Key Management Performance Metrics (30-Day Experiment, 200-Node Deployment).

Metrics	Value
Total key agreement attempts (aggregate, all proxies)	1247 times
Average agreement latency	42 ms
Maximum agreement latency	187 ms
Key update success rate	99.92%
Average key lifecycle	34.6 min
Total key renewals per node over 30 days	~1248 (theoretically)

Note: The 1247 total operations represent the aggregate count of all key agreements and updates across the entire 200-node deployment. The average key lifecycle of 34.6 min per node corresponds to approximately 1248 renewals per node over 30 days; the slight discrepancy (1247 vs. 1248) is due to rounding and the fact that not all nodes were active for the entire 30-day period.

**Table 10:** Cost comparison of three deployment schemes (200 nodes).

Cost Items	Pure QKD Scheme (Unit: CNY)	Pure PQC Scheme (Unit: CNY)	Tiered Scheme Proposed in This Work
Edge node hardware upgrades	¥150,000	¥40,000	¥10,000
QKD equipment and optical fiber	¥650,000	¥0	¥0
Proxy node deployment (10 units)	¥0	¥0	¥8000
Software development and integration	¥100,000	¥60,000	¥50,000
Total cost	¥900,000	¥100,000	¥68,000
Cost per node	¥4500	¥500	¥340

## Data Availability

The data presented in this study are available on request from the corresponding author. The data are not publicly available due to privacy and industrial confidentiality restrictions.

## References

[1] Chhetri R. (2026). Benchmarking NIST-Standardised ML-KEM and ML-DSA on ARM Cortex-M0+: Performance, Memory, and Energy on the RP2040. arXiv.

[2] Shor P.W. (1997). Polynomial-time algorithms for prime factorization and discrete logarithms on a quantum computer. SIAM J. Comput..

[3] Chhetri G., Somvanshi S., Hebli P., Brotee S., Das S. (2025). Post-quantum cryptography and quantum-safe security: A comprehensive survey. arXiv.

[4] Truong Q.D., Nguyen H., Nguyen T.T., Lee H. (2026). NIST post-quantum cryptography standards: A comprehensive review of theoretical foundations and implementations. IEEE Access.

[5] Houlès N., Heckmann T. (2026). Algorithmic Optimization of the Gaussian Sampler in the FN-DSA Post-Quantum Signature Scheme. Cryptol. ePrint Arch..

[6] FIPS 206 (2025). FN-DSA (Falcon)—Initial Public Draft[EB/OL].

[7] Botros L., Kannwischer M.J., Schwabe P. (2019). Memory-efficient high-speed implementation of Kyber on Cortex-M4. International Conference on Cryptology in Africa.

[8] Abdulrahman A., Hwang V., Kannwischer M.J., Sprenkels A. (2022). Faster kyber and dilithium on the cortex-m4. International Conference on Applied Cryptography and Network Security.

[9] Iskander R., Kirah K. (2026). Partial Number Theoretic Transform Masking in Post-Quantum Cryptography (PQC) Hardware: A Security Margin Analysis. arXiv.

[10] Sinaga R., Purnomo H.D., Sembiring I., Wellem T., Junaidi A. (2025). Adaptive Post Quantum Cryptography Tuning for Constrained Devices Using Kyber and Saber. Proceedings of the 2025 4th International Conference on Creative Communication and Innovative Technology (ICCIT).

[11] Nguyen T.T., Dao T.T., Luc N.Q. (2025). Develop a quantum key distribution application based on the BB84 protocol combined with a classical channel. Bull. Electr. Eng. Inform..

[12] Wang R., Gärtner J., Dubrova E. (2025). Decompressing dilithium’s public key with fewer signatures using side channel analysis. 2025 IEEE 55th International Symposium on Multiple-Valued Logic (ISMVL).

[13] Mahdi L.H., Abdullah A.A. (2025). Fortifying future IoT security: A comprehensive review on lightweight post-quantum cryptography. Eng. Technol. Appl. Sci. Res..

[14] Commey D., Appiah B., Klogo G.S., Bagyl-Bac W., Gadze J.D., Alsenani Y., Crosby G.V. (2025). Performance analysis and deployment considerations of post-quantum cryptography for consumer electronics. arXiv.

[15] Bormann C., Ersue M., Keranen A. (2014). Terminology for Constrained-Node Networks.

[16] Xie Y.D. (2025). Research and application of quantum encryption technology in optical communication systems. China Broadband.

[17] Grünenfelder F., Boaron A., Resta G.V., Perrenoud M., Rusca D., Barreiro C., Houlmann R., Sax R., Stasi L., El-Khoury S. (2023). Fast single-photon detectors and real-time key distillation enable high secret-key-rate quantum key distribution systems. Nat. Photonics.

[18] Karimi M., Shaefer R. (2025). IIoT communication protocols compatibility and security an in-depth review. TechRxiv.

[19] Cruz-Piris L., Marín-López A., Álvarez-Campana M., Sanz M., Moreno J.I., Arroyo D. (2025). Measuring the impact of post quantum cryptography in Industrial IoT scenarios. Internet Things.

[20] Sadeghi S., Cpi P. (2025). Secure and Efficient Signal Processing on FPGA: A Comprehensive Review of Cryptographic, Post-Quantum, and AI-Enhanced DSP Implementations for Embedded Systems. Int. J. Eng. Technol. Sci..

[21] Turan M.S., McKay K.A., Chang D., Kang J., Kelsey J. (2025). Ascon-based lightweight cryptography standards for constrained devices. NIST SP.

[22] Cirne A., Sousa P.R., Resende J.S., Antunes L. (2024). Hardware security for internet of things identity assurance. IEEE Commun. Surv. Tutor..

[23] Trungadi F., Fabiano M., Aloisio D., Brunaccini G., Sergi F., Merlino G., Longo F. (2025). Securing Modbus in legacy industrial control systems: A decentralized approach using proxies, Post-Quantum Cryptography and Self-Sovereign Identity. J. Inf. Secur. Appl..

[24] Bhowmick A., Saglani D., Maddali L.P., Rayala A., Dilip Thakur M.S., Mindigal Alasingara Bhattachar R. (2025). DoPQM: Devices Oriented Post-quantum Cryptographic Migration Strategies for an Enterprise Network. International Conference on Information Systems Security.

[25] Moon S.Y., Jo B.H., El Azzaoui A., Singh S.K., Park J.H. (2025). Edge-Fog Enhanced Post-Quantum Network Security: Applications, Challenges and Solutions. Comput. Mater. Contin..

[26] Villegas-Ch W., Gutierrez R., Sánchez-Salazar I., Mera-Navarrete A. (2024). Adaptive security framework for the internet of things: Improving threat detection and energy optimization in distributed environments. IEEE Access.

[27] Kannwischer M., Rijneveld J., Schwabe P., Stebila D., Wiggers T. (2019). PQClean: Clean, Portable, Tested Implementations of Post Quantum Cryptography. https://github.com/PQClean/PQClean.

[28] Jia J., Dong B., Kang L., Xie H., Guo B. (2023). Cost-optimization-based quantum key distribution over quantum key pool optical networks. Entropy.

[29] Kalnina E., Balodis R., Celms E., Kozlovics S., Opmane I., Petrucena K., Rencis E., Viksna J. (2025). Integration of PQC and QKD: Applications, Challenges and Implementation Frameworks. International Conference on Applied Cryptography and Network Security.

[30] Park J., Anandakumar N.N., Saha D., Mehta D., Pundir N., Rahman F., Farahmandi F., Tehranipoor M. (2022). PQC-SEP: Power Side-channel Evaluation Platform for Post-Quantum Cryptography Algorithms. Cryptol. ePrint Arch..

[31] Loke S.V. (2025). Evaluation of Lightweight Cryptography Algorithms of National Institute of Standards and Technology: Structural Design, Security Assessment and Implementation Performance. 2025 IEEE International Conference on Computation, Big-Data and Engineering (ICCBE).

[32] Thenmozhi A., Usha G. (2025). Balancing Quantum and Classical Security: Side-Channel Challenges in NIST PQC Finalist. 2025 3rd International Conference on Sustainable Computing and Data Communication Systems (ICSCDS).

[33] Hamarsheh A. (2024). An adaptive security framework for Internet of Things networks leveraging SDN and machine learning. Appl. Sci..

[34] Bisheh-Niasar M., Azarderakhsh R., Mozaffari-Kermani M. (2021). Instruction-set accelerated implementation of CRYSTALS-Kyber. IEEE Trans. Circuits Syst. I Regul. Pap..

[35] Bisheh-Niasar M., Karabulut E., Upadhyayula K., Norris M., Pillilli B. (2026). Adams Bridge Accelerator: Bridging the Post-Quantum Transition. Cryptol. ePrint Arch..

[36] Liang X., Qin Y., Pan S., Tan L. (2026). Constant-Time Symmetry Exploitation for NTT Twiddle Factors in ML-KEM: A Unified Cost Model for Embedded Deployments. Comput. Mater. Contin..

